# Joint Effects of Smoking and Silicosis on Diseases to the Lungs

**DOI:** 10.1371/journal.pone.0104494

**Published:** 2014-08-08

**Authors:** Lap Ah Tse, Ignatius T. S. Yu, Hong Qiu, Chi Chiu Leung

**Affiliations:** 1 JC School of Public Health and Primary Care, The Chinese University of Hong Kong, Hong Kong SAR, China; 2 Pneumoconiosis Clinic, Tuberculosis and Chest Service, Department of Health, Hong Kong SAR, China; MOE Key Laboratory of Environment and Health, School of Public Health, Tongji Medical College, Huazhong University of Science and Technology, China

## Abstract

Smokers are subject to being more susceptible to the long-term effects of silica dust, whilst it remains unclear whether the joint effect of smoking and silicosis differs amongst diseases to the lungs; this study aims to address this knowledge gap. This was a historical cohort study comprised of 3202 silicotics in Hong Kong during 1981–2005 who were followed up till 31/12/2006. We estimated the standardized mortality ratio (SMR) in the smoking and never smoking silicotics using the mortality rates of male general population indiscriminately by smoking status, but these SMRs were regarded as biased. We adjusted these biased SMRs using “smoking adjustment factors (SAF)”. We assessed the multiplicative interaction between smoking and silicosis using ‘relative silicosis effect (RSE)’ that was the ratio of SAF-corrected SMR of smoking silicotics to the never smokers. A RSE differs significantly from one implies the presence of multiplicative interaction. A significant excess SMR was observed for respiratory diseases (lung cancer, chronic obstructive pulmonary diseases [COPD], silicosis) and other diseases to the lungs (pulmonary heart disease, tuberculosis). All the ‘biased-SMRs’ in smokers were higher than those in never smokers, but the SAF-corrected SMRs became higher in never smokers. The RSE was 0.95 (95%CI: 0.37–3.55), 0.94 (95%CI: 0.42–2.60), and 0.81 (95%CI: 0.60–1.19) for lung cancer, COPD, and silicosis; whilst it was 1.21 (95%CI: 0.32–10.26) for tuberculosis and 1.02 (95%CI: 0.16–42.90) for pulmonary heart disease. This study firstly demonstrated the joint effect of smoking and silicosis may differ amongst diseases to the lungs, but power is limited.

## Introduction

Occupational epidemiological studies conducted in workers with silicosis have consistently shown increased risks of lung cancer and chronic obstructive pulmonary diseases (COPD) [1]–[3]; these increased risks, however, could be linked to their smoking behaviors because silicotic workers were generally more prone to cigarette smoking than the referent population. Yet, in the previous historical occupational cohort studies involving standardized mortality ratio (SMR), cigarette smoking was simply skeptics as a potential confounder to be adjusted or it was even not adjusted at all. Apart from being a possible confounder, smoking may interact with silica dust or the associated silicosis to modify the risk of multiple disease outcomes, in particular the diseases to the lungs, as the lungs are the target organs of hazardous substances (including cigarette smoke) whose route of entry of the body is by inhalation. Long-term cigarette smoking may damage the respiratory system [4], leading the smokers to being more susceptible than the never smokers to the adverse health effects due to the prolonged exposure to hazardous silica dust or the associated silicosis. It remains unclear whether the joint effect of smoking and silicosis differs amongst a variety of diseases to the lungs; this study aims to address this knowledge gap by using data from a historical cohort of Chinese workers with silicosis.

## Materials and Methods

### Cohort enumeration and follow-up

The cohort consists of 3202 male incident cases of silicosis diagnosed at the Pneumoconiosis Clinic of the Tuberculosis and Chest Service of the Department of Health (the only referral center for all patients with pneumoconiosis in Hong Kong) between 01/01/1981 and 31/12/2005, by adding 413 cases to the previous one [5]. The diagnosis of silicosis (profusion category 1/0 or higher) was made by a medical panel following the recommendation of the International Labor Organization [6], whereas the workers who had evidence of past exposure to asbestos were excluded. All eligible subjects were followed up till the end of 2006 and they contributed for a total of 36,890 person-years of observation, with a follow-up rate of 97.4%. Records of Chest Clinic, Pneumoconiosis Compensation Fund Board, and Death Registry were linked to determine the vital status for all cohort members and ascertain the causes of death during the observational period. The ICD (International Classification of Diseases, Ninth Revision) codes of causes of death for those who died were obtained from the Census and Statistics Department. Overall, 1562 deaths occurred during 1981–2006 and the underlying causes of death were ascertained for 1438 cases (92.1%).

The Survey and Behaviour Research Ethics Committee (SBREC) of the Chinese University of Hong Kong specially approved this study. Written informed consent from individual participant was exempted by the SBREC, and the patient records/information was anonymized prior to analysis.

### Exposure assessment of smoking and data collection

Each worker's baseline information (at the time of diagnosis of silicosis) on socio-demographics, smoking habit, lifetime occupational history, and medical history were obtained from the records of the Pneumoconiosis Clinic. In this study, we classified smoking habit as never and ever smoking (including either former or current smoking). An ever smoker was defined as one who had ever smoked more than 20 packs of cigarettes or 12 oz of tobacco in lifetime, or more than 1 cigarette a day or more than 1 cigars a week for 1 year [7]; otherwise, he was a never smoker. A former smoker was referred to an ever smoker who had quit smoking for two or more years at the time of baseline survey, otherwise, an ever smoker was defined as a current smoker. Lifetime occupational history included age at first exposure to silica dust, occupation and industry of each work episode, and net years of exposure to silica dust.

### Standardized mortality ratio (SMR) and the correction

Expected number of deaths for each cause was calculated by multiplying the age-period-specific person-years at risk by the corresponding mortality rates of the entire Hong Kong male general population, while the SMR and the 95% confidence interval (95% CI) were estimated under the assumption of a Poisson distribution for the observed deaths [8]; however, using the mortality rate in the referent population without a separation of smokers from never smokers might result in an artificial underestimation of SMR for the never smoking silictoics and an overestimation in the smoking group [9]. We corrected these ‘biased-SMRs’ in smoking and never smoking silicotics using a validated method of ‘smoking adjustment factor (SAF)’ [9], as shown in Table 1; while the calculation of SAF is illustrated in Supplement S1.

**Table 1:** Summary of smoking adjustment factors (SAF) and the population attributable fraction (PAF) due to smoking for the major causes of deaths to the lungs.

Causes of death	OR (95% CI for ever	Population	Smoking adjustment	factors (SAF)^d^
	smokers^a^	attributable fraction	Never smokers	Ever smokers
Lung cancer	4.99 (4.00–6.22)	0.672	3.05	0.61
Chronic obstructive pulmonary diseases	3.68 (2.58–5.26)	0.579	2.37	0.65
Silicosis^b^	1.80 (1.20–2.60)	0.294	1.42	0.78
Pulmonary heart disease^c^	1.78 (1.36–2.33)	0.286	1.40	0.79
Pulmonary tuberculosis	2.54 (1.24–5.22)	0.441	1.79	0.70

Abbreviations: OR, odds ratio (ever smoking vs. never smokers); 95% CI, 95% confidence interval.

a All the odds ratios other than silicosis for smoking (ever vs. never smoking) was obtained from a case-control study of all adult deaths in Hong Kong [17].

b We assumed Hong Kong male smokers having the same risk of silicosis as those of Italy ceramic workers; however, we had to use the incidence OR for silicosis (smoking vs. never smoking) to represent the mortality OR, as we do not have relevant mortality data in Hong Kong [18].

c The odds ratio of other vascular disease was used to estimate the SAF-corrected SMR for pulmonary heart disease [17].

d The SAF for smoking silicotics was numerically equal to 1/[(1-PAR%)RR] and that for nonsmoking silicotics was 1/(1-PAR%) (detailed calculations were illustrated in *Supplement S1*).

### Assessment of interaction (or effect modification) between silicosis and smoking

We examined multiplicative interaction between silicosis and smoking on specific causes of death using the approach of ‘relative silicosis effect (RSE)’ by comparing the SAF-corrected SMR of smoking silicotics to that of the never smokers [9]. We calculated the 95% CI of the RSE based on the maximum likelihood method described by Breslow and Day [10]. If the RSE significantly differs from 1, it implies that smoking is likely to be an effect modifier (e.g., if RSE>1, it suggests that the effect of silicosis on mortality among smokers was higher than the never smokers) and a separate report on the SAF-corrected SMR for the subgroup of smoking and never smoking silicotics should be delivered. Alternatively, if the RSE is not statistically significant, it suggests the absence of multiplicative interaction, and smoking in this context is likely to be a confounding factor which would have to be adjusted accordingly. We compared the smoking indirectly adjusted “SAF-corrected SMR” using the method proposed by Axelson [11] and the consistent findings (Supplement S2) suggested that the methodology of this SAF method is valid. One advantage of using SAF method is that it could not only be used to evaluate the multiplicative interaction between smoking and silicosis but also to indirectly adjust for the potential confounding effect of smoking in studies of SMR.

## Results

### The description of cohort

The mean age at diagnosis of silicosis was 55.3 (S.D.: 10.5) years and the deceased died at 66.5 (S.D.: 10.6) years old after an average of 11 years of follow-up. Most workers (93%) migrated from mainland China and 49% had a history of tuberculosis. Large opacities were seen in 649 (20.3%) workers at the time of diagnosis of silicosis. There were 2858 (89.3%) ever smokers (current smokers: 52.7%; former smokers: 47.3%) and 327 (10.2%) were never smokers, while smoking data were missing in 17 subjects (0.53%). About a half of the workers (50.2%) had first exposure to silica dust at age of 15–24 years. There were 1629 (50.8%) surface construction workers, 1210 (37.8%) underground caisson workers, and 363 (11.3%) workers employed in other dusty trades (e.g., tunneling, mining, and metal manufacturing). Overall, the mean net years of silica dust exposure was 24.7 (S.D.: 9.6) years, which was about 2.5 years shorter than the average years of cigarette smoking.

Among all 3202 silicotic workers, a significantly excess risk of death was observed for all causes (SMR = 2.33, 95% CI: 2.22–2.45), diseases from respiratory system (including lung cancer, chronic obstructive pulmonary diseases [COPD], silicosis) and other diseases to the lungs (pulmonary heart disease, pulmonary tuberculosis) (Table 2). Table 3 shows that all the ‘biased-SMRs’ in smokers were higher than those in the never smokers. Compared with the ‘biased-SMRs’, the SAF-corrected SMRs decreased substantially in the smoking subgroup but they increased consistently in the never smoking subgroup. Except for the pulmonary tuberculosis and heart disease, never smoking silicotics tended to have higher SAF-corrected SMRs than the smoking silicotics for respiratory diseases (i.e., lung cancer, COPD, and silicosis), though some SAF-corrected SMRs in never smokers were not statistically significant due to a small number of deaths. The SAF-corrected SMR for all causes of death among smoking and never smoking silicotics was 1.85 (95% CI: 1.75–1.95; 1425 deaths) and 2.47 (95% CI: 2.07–2.94; 125 deaths), respectively.

**Table 2:** Numbers of observed and expected deaths and the standardized mortality ratios (SMR) for major causes of deaths to the lungs among 3202 male silicotic workers in Hong Kong, 1981–2006.

Causes of death	ICD-9^th^ code	Observed	Expected	SMR (95% CI)
Lung cancer	162	157	84.27	1.86 (1.59–2.17)
Chronic obstructive pulmonary diseases	490–496	172	58.18	2.96 (2.55–3.43)
Silicosis	502	510	1.01	504.95 (462.99–550.72)
Pulmonary heart disease	415–417	18	3.45	5.22 (3.30–8.25)
Pulmonary tuberculosis	011	52	7.92	6.57 (5.01–8.61)

Abbreviations: OR, odds ratio; 95% CI, 95% confidence interval.

**Table 3:** Relative silicosis effect, the biased and SAF-corrected standardized mortality ratio (SMR) for major causes of death to the lungs in smoking and never smoking silicotics.

Causes of death	No. of deaths^a^	Biased-SMR		SAF-corrected SMR		Relative silicosis effect
		Never smokers	Smokers	Never smokers	Smokers	
Lung cancer	4/152	0.43 (0.17–1.10)	2.04 (1.74–2.39)	1.31 (0.51–3.37)	1.25 (1.06–1.46)	0.95 (0.37–3.55)
Chronic obstructive pulmonary diseases	6/164	0.93 (0.43–2.02)	3.20 (2.75–3.73)	2.20 (1.01–4.81)	2.07 (1.77–2.41)	0.94 (0.42–2.60)
Silicosis	39/467	354.55 (259.37–484.64)	518.89 (473.91–568.13)	499.36 (365.31–682.60)	405.38 (370.24–443.85)	0.81 (0.60–1.19)
Pulmonary heart disease	1/16	2.86 (0.50–16.19)	5.19 (3.20–8.44)	4.02 (0.71–22.80)	4.09 (2.52–6.65)	1.02 (0.16–42.90)
Pulmonary tuberculosis	2/50	2.33 (0.64–8.48)	7.13 (5.41–9.40)	4.16 (1.14–15.18)	5.03 (3.81–6.63)	1.21 (0.32–10.26)

Abbreviations: SAF, smoking adjustment factor.

(#) in parenthesis is 95% confidence interval of SMR or relative silicosis effect.

a Number of deaths in never smokers to ever smokers.

Table 3 also shows the RSEs for the major causes of death from respiratory diseases and other diseases to the lungs. The RSE was 0.95, 0.94, and 0.81 for lung cancer, COPD, and silicosis, which indicates that the effect (risk ratio) of silicosis on the mortality from these respiratory diseases in never smokers was relatively stronger than the smokers. The RSE was 1.21 for the pulmonary tuberculosis, which suggests that the risk ratio effect of silicosis on pulmonary tuberculosis was relatively weaker in never smokers. A close to null RSE (1.02) for the pulmonary heart disease implies the role of smoking of being a likely confounding factor to be adjusted for the association between silicosis and pulmonary heart disease, and hence, smoking being a potential confounder may have explained 21.11% (5.22/4.31 - 1) of the risk of deaths from the pulmonary heart disease among silicotic workers.

## Discussion

We found that workers with silicosis were associated with an increased risk of mortality from all causes and the respiratory diseases (i.e., lung cancer, COPD, silicosis), with a relatively stronger risk ratio effect of silicosis in never smokers given the RSE of lower than one. An excess risk of death was also observed from pulmonary tuberculosis, but the silicosis effect was relatively weaker in never smokers (i.e., the RSE was higher than one), which indicates a positive multiplicative interaction may exist between smoking and silicosis on the mortality from pulmonary tuberculosis. These results suggest that cigarette smoking may interact with silicosis to modify the risks of different types of diseases to the lungs in a different way; hence, a better understanding of the role of smoking in the etiology pathway of diseases to the lungs and the biological mechanisms behind is of important scientific values in designing effective measures of control against diseases to the lungs among workers with silicosis.

We corrected the biased SMR of lung cancer and other diseases to the lungs for smokers and never smokers using a validated approach of SAF. This is merit because none of the previous occupational cohort studies of silica or silicosis using the general population as the external referent group had attempted to correct the biased SMR in the smoking and never smoking subgroups. The SMR is subject to be underestimated in never smokers but overestimated in smokers if the mortality rates of general population applied indiscriminately [9]. After the “biased-SMR” was corrected by the SAF, we observed a 31% increase in lung cancer mortality among never smoking silicotics; such finding was in concordance with two recent population-based case-control studies in Europe and China in which a similar independent effect of silica on lung cancer risk was observed among never smoking men [12], [13]. Moreover, a previous meta-analysis revealed a significant RSE of 0.29 (95% CI: 0.20–0.42) between smoking and silicosis on lung cancer risk after combining 10 cohort mortality studies of silicosis [9]. The relatively weaker RSE observed in our cohort study (0.95, 95% CI: 0.37–3.55) than that of the meta-analysis could be explained by a less selection bias from our cohort study. Compared with other compensation-based studies where lung impairment was generally indicated, we have less symptomatic silicotics in the cohort, because in Hong Kong, compensation would be paid out for all confirmed cases of silicosis regardless of any indication of lung impairment. On the other hand, the weaker RSE obtained in this study may partly be due to the use of ILO 1/0 as diagnostic criteria for silicosis, rather than the standard ILO 1/1.

An increased SAF-corrected SMR was suggested for COPD mortality among our smoking (2.07) and never smoking silicotics (2.20), yielding a RSE of 0.94; meanwhile, a slightly greater RSE of 0.81 was demonstrated for the death from silicosis. Given a consistent pattern of RSEs from the respiratory diseases that were less than “one”, our study indicated that cigarette smoking may act as an effect modifier to yield a negative (risk-ratio) multiplicative interaction for the association between silicosis and the respiratory diseases from lung cancer, COPD, and silicosis.

The apparently higher mortality rate ratio from the respiratory diseases (lung cancer, COPD, silicosis) among never smoking silicotics than the smoking silicotics (expressed as a RSE<1) could be explained by the ‘harvesting’ of genetically predisposed subjects by smoking, or that smoking to be a stronger risk factor competed with other environmental toxins of relatively low potency (e.g., silica) to induce deaths in the susceptible workers. It was likely that a higher proportion of deaths from lung diseases occurred in smoking silicotics that would be attributable to smoking (being harvested by smoking) and hence leaving only a few susceptible individuals for silica/silicosis to exert their effects. On the other hand, more silicotics who had never smoked remained susceptible to the agents of relatively weaker toxicity (e.g., silica), leading the adverse effect of silica/silicosis among never smokers to being more marked than those among the smokers.

Cigarette smoking was found to be associated with an increased risk of active tuberculosis in cohort studies of general population [14], [15], whilst its role in the occupational cohort studies of workers with silicosis has not been documented. We observed a slightly higher excess mortality of pulmonary tuberculosis among smoking silicotics (SAF-corrected SMR = 5.03) than the never smokers (SAF-corrected SMR = 4.16), with a RSE of 1.21; this suggests that cigarette smoking may interact with silicosis to enhance the adverse effect of silicosis on the mortality from pulmonary tuberculosis (i.e., positive multiplicative interaction) in a different way from that of the respiratory diseases observed in our study. Cigarette smoking has pro-inflammatory and immunosuppressive effects that have been evident to worsen the host response to infectious agents [16], while the immune responses may be further suppressed by the direct toxic effects of silica dust and/or the secondary chronic inflammatory lung fibrosis that could lead the smoking silicotic workers to a greater chance of contracting tuberculosis than the never smokers. Despite the current study was in lacking of power to achieve a statistical significance due to an inadequate number of cases in never smokers, the interactive effect studied here demonstrated a novel area that was not investigated in previous occupational cohort studies, and hence has added newly valuable evidence to the current literature.

Sensitively analysis based on different prevalence of smoking among Hong Kong male general population shows that the RSE for different lung diseases remains stable, which indicates robustness of the methodology for the calculation of SAF and RSE. However, there are concerns from the subgroup of 124 deceased with unknown cause of death (7.9%) but this limitation may lead the risk estimate toward the null as there was no obvious difference in the distributions of occupational exposure and demographic information between these two subgroups. We realized that we do not have information on when the subjects started and stopped smoking, which made us not possible to assess the temporal relationship amongst the start of smoking, first exposure to dust, and development of silicosis; nevertheless, limited information obtained from our study showed that smoking behavior may start on an average of 2.5 years ahead of the dust exposure among our silicosis patients in Hong Kong, given a shorter duration of silica dust exposure than the years of smoking. Since there is no local population data on the separate mortality rates of specific cause of death for the subgroup of current and former smokers, the assessment on the multiplicative interaction and the indirect adjustment were only carried out for the ever smokers [17] and this point has posed another limitation to the current study.

In conclusion, this study demonstrated that the independent risk ratio effect of silicosis on the respiratory diseases (including lung cancer, COPD, and silicosis) among smokers was 81–95% of that among the never smokers, whilst the risk ratio effect of silicosis on the pulmonary tuberculosis in smokers was oppositely 21% higher than that in the never smokers. Cigarette smoking may have different joint effect with silicosis on a variety of diseases to the lungs. Due to limited power, results from this historical cohort study would have to be confirmed by larger studies and in other populations.

## Supporting Information

Supplement S1 Calculation of an innovative ‘smoking adjustment factors (SAF)’ for standardized mortality ratio (SMR) in the subgroups of smokers and never smokers.
(DOCX)

Supplement S2 **Comparing the method of ‘smoking adjustment factors (SAF)’ with the Axelson's indirect method using pulmonary heart disease as an example.**
(DOCX)
