# Supplementary material for: Joint Effects of Smoking and Silicosis on Diseases to the Lungs
Source: PLoS One. 2014 Aug 8;9(8):e104494. doi: 10.1371/journal.pone.0104494 (PMC4126694; doi:10.1371/journal.pone.0104494)
Supplement: Supplement S1 — Calculation of an innovative ‘smoking adjustment factors (SAF)’ for standardized mortality ratio (SMR) in the subgroups of smokers and never smokers. (DOCX) [file pone.0104494.s001.docx]

Supplement 1. Calculation of an innovative ‘smoking adjustment factors (SAF)’ for standardized mortality ratio (SMR) in the subgroups of smokers and never smokers

We calculated the population attributable fraction (PAF) for smoking for the selected cause of death in Hong Kong male general population according to the relative risk (RR) of smoking and the smoking prevalence (P_s_) in the general population: PAF=P_s_(RR-1)/[1+P_s_ (RR-1)]. During the study period, the average P_s_ for the male general population in Hong Kong for the period 1981-1999 was 51.3% (the source of smoking prevalence among Hong Kong male population was obtained from the Hong Kong Census and Statistics Department). By definition, PAF=(I_p_-I_ns_)/I_p_, where I_p_ and I_ns_ are the mortality rates in the total population and among never smokers. Therefore, I_ns_=I_p_(1-PAF) and the mortality rate among ever smokers (I_s_)=I_p_(1-PAF)RR. Since the expected numbers of a selected cause of death (e.g., pulmonary heart disease) among never smokers (E_ns_) in our cohort of silicotic workers were overestimated by using I_p_ instead of I_ns_, it should be adjusted by a factor of (1-PAF) and the corresponding SMR should be adjusted by a ‘smoking adjustment factor (SAF)’ of 1/(1-PAF). Likewise, the SAF for SMR among smokers would be 1/[(1-PAF)RR].

Using pulmonary heart disease as an example:

Given RR=1.78, P_s_=51.3%.

Then, PAF=P_s_(RR-1)/[1+P_s_(RR-1)]=0.513(1.78-1)/[1+0.513(1.78-1)]=28.6%.

Hence, I_n_=I_p_(1-PAF)=I_p_(1-0.286)=0.714I_p_, and I_s_=I_p_(1-PAF)RR=I_p_(1-0.714)*1.78=0.51I_p_.

According to the above formula, the SAF to correct the ‘biased SMR’ for the never smokers is 1/(1-PAF)=1/(1-0.286)=1.40; the SAF to correct the ‘biased SMR’ for the ever smokers is 1/[(1-PAF)RR]=1/[(1-0.286)*1.78]=0.79.
