# Supplementary material for: Joint Effects of Smoking and Silicosis on Diseases to the Lungs
Source: PLoS One. 2014 Aug 8;9(8):e104494. doi: 10.1371/journal.pone.0104494 (PMC4126694; doi:10.1371/journal.pone.0104494)
Supplement: Supplement S2 — Comparing the method of ‘smoking adjustment factors (SAF)’ with the Axelson's indirect method using pulmonary heart disease as an example. (DOCX) [file pone.0104494.s002.docx]

Supplement 2. Comparing the method of ‘smoking adjustment factors (SAF)’ with the Axelson’s indirect method using pulmonary heart disease as an example

Below demonstrates the Axelson's method for the indirect adjustment of potential confounding effect of smoking for pulmonary heart disease:

|  | Equation and Estimation | Notation |
| --- | --- | --- |
| Study cohort | I_S_ = I_CF_ * P_CF_ + I_0_ (1-P_CF_)  = RRI_0_ * P_CF_ + I_0_ (1-P_CF_ )  = (1.78)(I_0_)(0.8927)+ I_0_(0.1073)  = 1.70 I_0_ | Is = death rate from pulmonary heart disease in the study cohort;  RR was the relative risk of pulmonary heart disease for smokers vs. never smokers among Hong Kong males (1.78);  P_CF_ (for ever smokers) in this cohort was 89.27% |
| General population in Hong Kong | I_G_ = (1.78)(I_0_)(0.513)+I_0_(1-0.513)  = (1.78)(I_0_)(0.513)+I_0_(0.487)  = 1.40 I_0_ | I_G_ = death rate from pulmonary heart disease in the Hong Kong male general population;  P_CF_ in Hong Kong male general population in the corresponding age groups was 51.3% during the study period; |

Hence, the relative risk of pulmonary heart disease in this cohort due to the higher prevalence of smokers compared to the Hong Kong male general population is: RR=I_S_/I_G_=1.70I_0_/1.40I_0_=1.21. Hence, the smoking indirectly adjusted SMR for pulmonary heart disease using Axelson's indirect method among our silicotic cohort was 4.31 (5.22/1.21=4.31).

Below shows the method of ‘smoking adjustment factor’ to indirectly adjust for the potential confounding effect from smoking for the ‘crude’ SMR:

According to the ***Supplement 1***, a factor to adjust for the ‘biased expected number of deaths’ among never smoking silicotics is (1-PAF) and the corresponding factor among ever smoking silicotics is 1/[(1-PAF)RR]. We calculated the smoking indirectly adjusted SMR for pulmonary heart disease according to the method of ‘smoking adjustment factor’ (see below), and found that the result was exactly same as that of the Axelson’s indirect method.

Adjusted SMR = O/ (E_ns_ + E_s_) = O / [E*(1-P_s_)*(1-PAF)+P_s_*(1-PAF)*RR]

=Crude SMR / [(1-P_s_)*(1-PAF) + P_s_ * (1-PAF)*RR]

=5.22 / [(1-0.8927)*(1-0.286) + 0.8927*(1-0.286)*1.78]

=5.22 / (0.1073*0.714+0.8927*0.714*1.78)

=5.22/ 1.21

=4.31

where, the E_ns_, E_s_, and E is the expected number of death from pulmonary heart disease in never smoking silicotics, ever smoking silicotics, and entire cohort of silicotics.
